# Molecular subtyping of bladder cancer using Kohonen self-organizing maps

**DOI:** 10.1002/cam4.217

**Published:** 2014-08-20

**Authors:** Edyta M Borkowska, Andrzej Kruk, Adam Jedrzejczyk, Marek Rozniecki, Zbigniew Jablonowski, Magdalena Traczyk, Maria Constantinou, Monika Banaszkiewicz, Michal Pietrusinski, Marek Sosnowski, Freddie C Hamdy, Stefan Peter, James WF Catto, Bogdan Kaluzewski

**Affiliations:** 1 Department of Clinical Genetics, Medical University of Lodz3 Sterlinga Street, Lodz, 91-425, Poland; 2 Institute for Cancer Studies and Academic Urology Unit, University of SheffieldBeech Hill Road, Sheffield, S10 2RX, UK; 3 Department of Ecology and Vertebrate Zoology, Faculty of Biology and Environmental Protection, University of Lodz12/16 Banacha Street, Lodz, 90-237, Poland; 4 Division of Urology, John Paul II Memorial Regional Hospital in Belchatow123 Czapliniecka Street, Belchatow, 97-400, Poland; 5 NZOZ Urological Doctors, “Marek Rozniecki and Partners”62 Warszawska Street, Lask, 98-100, Poland; 6 1st Clinic of Urology, Medical University of Lodz113 Zeromskiego Street, Lodz, 90-549, Poland; 7 Nuffield Department of Surgical Sciences, University of OxfordOld Road Campus Research Building, Oxford, OX3 7DQ, UK

**Keywords:** Bladder cancer, Kohonen self-organizing map, molecular markers, progression

## Abstract

Kohonen self-organizing maps (SOMs) are unsupervised Artificial Neural Networks (ANNs) that are good for low-density data visualization. They easily deal with complex and nonlinear relationships between variables. We evaluated molecular events that characterize high- and low-grade BC pathways in the tumors from 104 patients. We compared the ability of statistical clustering with a SOM to stratify tumors according to the risk of progression to more advanced disease. In univariable analysis, tumor stage (log rank *P* = 0.006) and grade (*P* < 0.001), HPV DNA (*P* < 0.004), Chromosome 9 loss (*P* = 0.04) and the A148T polymorphism (rs 3731249) in *CDKN2A* (*P* = 0.02) were associated with progression. Multivariable analysis of these parameters identified that tumor grade (Cox regression, *P* = 0.001, OR.2.9 (95% CI 1.6–5.2)) and the presence of HPV DNA (*P* = 0.017, OR 3.8 (95% CI 1.3–11.4)) were the only independent predictors of progression. Unsupervised hierarchical clustering grouped the tumors into discreet branches but did not stratify according to progression free survival (log rank *P* = 0.39). These genetic variables were presented to SOM input neurons. SOMs are suitable for complex data integration, allow easy visualization of outcomes, and may stratify BC progression more robustly than hierarchical clustering.

## Introduction

Bladder cancer (BC) is a common disease for which the outcomes have not improved in the last three decades 1. This probably reflects a lack of community-based screening for the disease, that advanced BC responds poorly to chemotherapy and that it can be hard to judge the need for radical treatment in patients with non-muscle invasive (NMI) disease. The latter arises primarily from a lack of knowledge regarding the biology of this disease. Clinicopathological and molecular data suggest two distinct pathways of urothelial carcinogenesis 2,3. Low-grade NMI cancers arise through regional deletion of chromosome 9, mutation of *FGFR3* (Fibroblast growth factor receptor), and *H-RAS* 4. High-grade tumors may present with or before the onset of muscle invasion and are best characterized by loss of (Tumor Protein) function through direct (e.g., mutation or deletion of *TP53*) or indirect (e.g., loss of *RB1*—Retinoblastoma or upregulation of *MDM2*—Murine Double Minute) means 5. High-grade tumors also have widespread chromosomal instability (polysomy, aneuploidy) and numerous changes to their epigenome 6,7.

While the two-pathway biology of BC is generally accepted, many tumors have aspects of low- and high-grade biology. For example, *FGFR3* mutations are not found in CIS (carcinoma in situ) but they coexist with *TP53* mutations in 10–20% of invasive BCs as do deletions of both chromosome 9 (typical of low-grade disease) and 17p (locus of *TP53*) in 15–74% BC 4,8. Clinical phenotypes, therefore, reflect either the timing or impact of genetic events combined with patient factors (such as type and continued exposure to carcinogens) and treatment effectiveness (such as timing, appropriateness and quality of treatment). A current challenge for translational researchers is to integrate distinct and, potentially, competing molecular events into single-phenotype predictions. In BC, this represents the ability to discriminate future tumor behavior using molecular alterations typical for low- and high-grade tumor development. Nonstatistical methods are appealing in this role as they do not rely upon data distribution, can handle large datasets automatically without supervision or prior assumptions, and do not assume that statistical proximity equates to molecular association 9. Various structures of artificial intelligence have been developed, of which Artificial Neural Networks (ANNs) are perhaps the best evaluated (reviewed in Ref. 10). Here, we report the use of a self-organizing map (SOM) to integrate molecular parameters in BC. SOMs are a type of unsupervised ANNs that are good for low-density data visualisation 11. We selected molecular events that characterize high- and low-grade BC pathways and used progression to more advanced disease as our primary outcome.

## Materials and Methods

### Patients, tumors, and samples

A total of 104 patients with BC were studied in this report (data in Table 1). The tumors were chosen at random to represent the disease spectrum from three Departments of Urology located in Lodz Macroregion, where the textile industry was very popular in the previous century. Tumors were graded according to 1973 WHO classification and staged using the TNM criteria 12. This study was approved by the ethics committee of the Medical University of Lodz (No: RNN/99/11/KE) and all patients gave written informed consent before entry.

**Table 1:** Genetic tests results, recurrence, and progression rate

	Overall *n* (%)	Kaplan–Meier analysis			
	
		Recurrence		Progression	
		
		Rate	Log–rank value	Rate	Log–rank value
Total	104 (100)				
*CHEK2* mutation					
Yes	7 (6.7)	2	*P* = 0.858	2	*P* = 0.250
No	97 (93.3)	22		13	
*FGFR3* mutation					
Yes	39 (37.5)	10	*P* = 0.618	5	*P* = 0.772
No	65 (62.5)	14		10	
*TP53* mutation					
Yes	15 (14.4)	5	*P* = 0.291	11	*P* = 0.160
No	89 (85.6)	19		4	
TP53 expression					
Altered	29 (27.9)	2	*P* = 0.858	4	*P* = 0.924
Normal	75 (72.1)	22		11	
Chromosome 9					
LOH	33 (31.7)	5	*P* = 0.229	5	*P* = 0.743
No	71 (68.3)	19		10	
Chromosome 13					
LOH	6 (5.8)	0	*P* = 0.740	1	*P* = 0.740
No	98 (94.2)	24		14	
Chromosome 17					
LOH	12 (11.5	4	*P* = 0.405	2	*P* = 0.978
No	92 (88.5)	20		13	
UroVysion test					
Positive	74 (71.2)	16	*P* = 0.414	11	*P* = 0.930
Negative	30 (28.8)	8		4	
*CDKN2A* polymorphism					
148G/A	7 (6.7)	0	*P* = 0.298	2	*P* = 0.298
148G/G	97 (93.3)	24		13	
*CYP1B1* polymorphism					
355T/T	14 (13.5)	4	*P* = 0.973	5	*P* = 0.417
355G/T	46 (42.2)	10		9	
355G/G	44 (55.7)	10		10	
*TP53* polymorphism					
72G/G	55 (52.9)	10	*P* = 0.106	10	*P* = 0.360
72G/C	41 (39.4)	10		5	
72C/C	8 (7.7)	4		0	
HPV DNA detected					
Yes	14 (13.5)	3	*P* = 0.975	1	*P* = 0.02
No	90 (86.5)	21		13	

### RNA and DNA extraction

RNA and DNA were extracted from bladder tumors, peripheral blood, and urinary sediments. For tumors, frozen tissues were homogenized in TRI REAGENT (guanidine thicyante/phenol, Molecular Research Center, Inc. cat. No TR-118) using ceramic beads (Roche MagNA Lyser Green Beads, Roche Applied Science, Mannheim, Germany, cat. No 3358941001) and the Roche Magna Lyser (cat. No 03 358 976 001: three times for 45 sec at 9283 g). Homogenized samples were cooled and RNA isolated by acid guanidinum thiocyanate-phenol-chloroform extraction (according to the manufacturer's protocol) 13. Total RNA was eluted in nuclease-free water and stored at −70°C. RNA was assessed for degradation, purity, and DNA contamination by spectrophotometry and electrophoresis in 1.0% ethidium bromide-stained agarose gel. Total RNA was DNase treated using DNase I (RNase free) reagent (Ambion, Life Technologies Polska Sp. z o.o., Warsaw, Poland, cat. No AM2222). First-strand cDNAs were synthesized from equal amounts of total RNA (0.5 *μ*g/reaction) using oligo(dT) and iScript cDNA Synthesis Kit (Bio-Rad Polska Sp. z o.o., Warsaw, Poland, cat. No 70-8890) according to the manufacturer's instruction. For blood samples, Roche MagNA Pure Compact automatic workstation was used to isolate DNA (Nucleic Acid Isolation Kit I-Large Volume, cat. No 03 730 972 001, Roche Diagnostics GmbH, Mannheim, Germany). For exfoliated urinary cells, 200–400 mL of freshly voided urine was collected in Carbowax at diagnosis. DNA was isolated from urine sediment with a Sherlock AX Kit according to manufacturer's guidelines (A&A Biotechnology s.c., Gdynia, Poland).

### Quantitative polymerase chain reaction

*TP53* expression was measured using quantitative polymerase chain reaction (qPCR) performed using an iCycler iQ System (Bio-Rad cat. No 170-8701, 1709750) 14. Expression was determined SYBR Green I fluorescence and normalized with respect to *GAPDH* (Glyceraldehyde-3-Phosphate Dehydrogenase) and *HPRT* (Hypoxanthine–guanine Phosphoribosyltransferase) genes.

### Mutation and deletion detection

Mutations in *TP53* (exons 4–8), *CDKN2A* (Cyclin-Dependent Kinase inhibitor *2A*, exons 1*α*, 2, and 3), and *FGFR3* (exons 7, 10, 15) were detected using single strand conformational polymorphism (SSCP) analysis and Sanger sequencing, as detailed 15–17. The mutations in *CHEK2* (Chekpoint Kinase, IVS2 + 1G>A, 1100delC, and I157T) gene were detected using multiplex PCR 18. Loss of heterozygosity (LOH) for the *TP53*,*/ARF,* and *RB1* genes was studied using PCR technique with malignant and wild-type (blood, genomic) DNA 19.

### UroVysion test

The UroVysion (Vysis) test consists of a four-color, four-probe mixture of DNA probe sequences homologous to specific regions on chromosomes 3, 7, 9, and 17, and was carried out according to the manufacturer's protocol.

### Human papilloma virus detection

Human Papilloma Virus (HPV) DNA was detected using the LINEAR ARRAY Human Papillomavirus GENOTYPING Test in cancer tissue (Roche, includes 37 pathogenic genotypes: 6, 11, 16, 18, 26, 31, 33, 35, 39, 40, 42, 45, 51, 52, 53, 54, 55, 56, 58, 59, 61, 62, 64, 66, 67, 68, 69, 70, 71, 72, 73, 81, 82, 83, and 84) according to manufacturer's protocol.

### Generation of a self-organizing map

The dataset (10 genetic variables × 104 patients) was presented to 10 input neurons seven times in the rough-training phase and 27 times in the fine-tuning phase. The number of the input neurons was equal to the number of variables in the dataset. On a basis of the established link between the input and output neurons, a virtual patient (in terms of values of the genetic variables presented to the SOM) was created in each output neuron. The output neurons were arranged on a two-dimensional grid (4 × 4). To cluster the virtual patients (and respective output neurons), the hierarchical cluster analysis with the Ward linkage method and Euclidean distance measure was used 20–22. Finally, each real patient was assigned to the best matching virtual patient and the respective output neuron. The SOM training process was performed with the use of the SOM Toolbox developed by the Laboratory of Information and Computer Science in the Helsinki University of Technology (http://www.cis.hut.fi/projects/somtoolbox/) in Matlab environments 23,24. The significance of differences between subclusters was assessed: 1) with the Tichý and Chytrý analysis and the Monte Carlo randomization test carried out with PC-ORD software for binary variables, and 2) with the Kruskal–Wallis test and the post hoc Dunn test for the variables measured at the ordinal or ratio level (STATISTICA Vsn. 10, 2011, StatSoft Polska Sp. z o.o., Krakow, Poland) 25.

### Statistical data analysis

**Figure 1:**
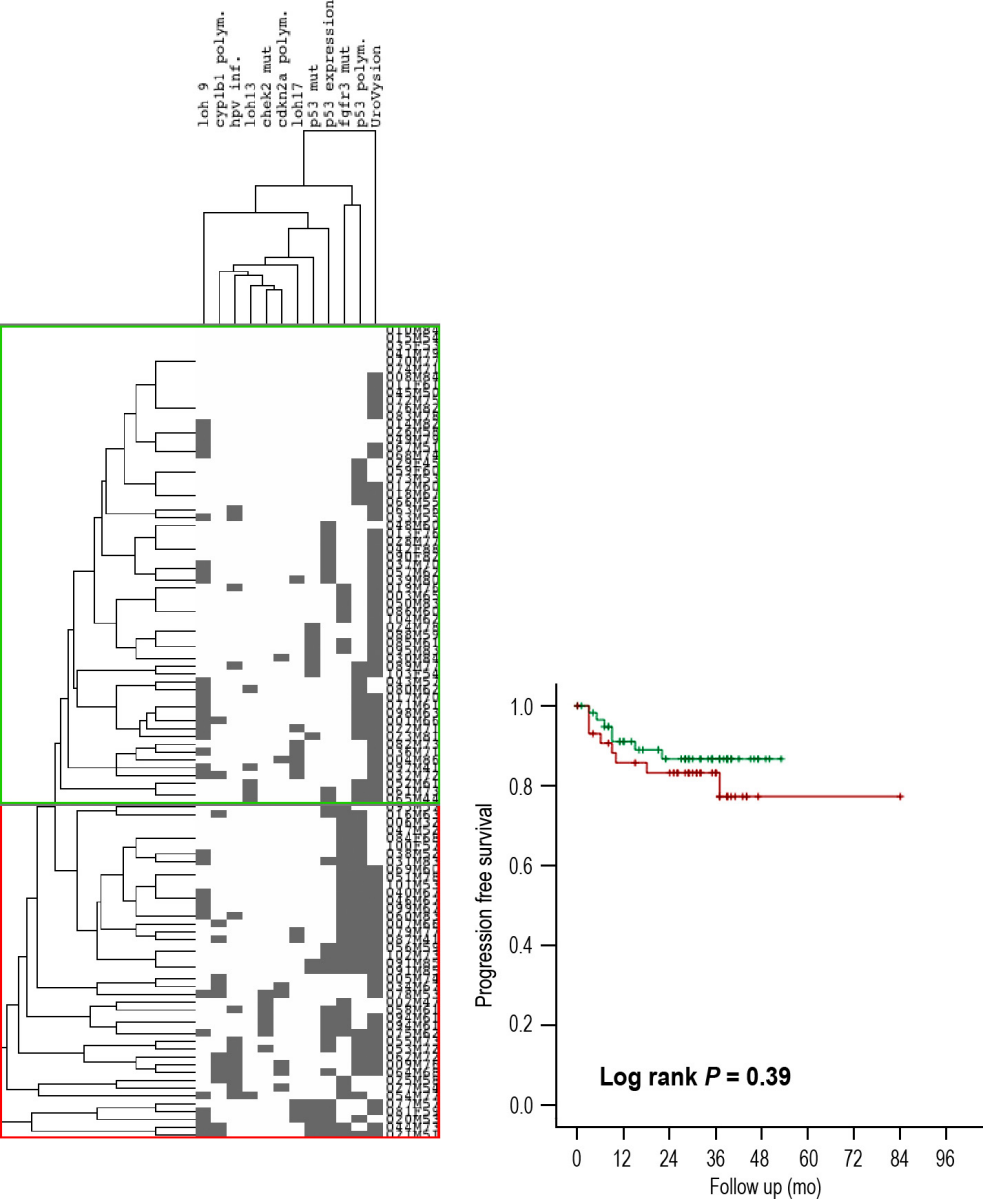
Hierarchical clustering of samples.

The primary aim of our study was to evaluate the ability of the SOM at integrating molecular data from BC samples. To this end, we analyzed its ability to stratify tumor progression using log-rank analysis and by plotting survival using the Kaplan–Meier method (SPSS Vsn. 19.0, IBM Inc., New York, NY) (Fig.1). Progression was defined as pathological or radiological evidence or worsening tumor stage. The most common examples were the development of muscle invasion from a NMI tumor, and or metastases from an invasive cancer. Both these events mark a significant deterioration in prognosis for the patient and a need to alter treatment intent. For comparison with the SOM, we used an unsupervised hierarchical approach to cluster tumors using city block distance and average linkage in Cluster 3.0 (Eisen Lab, University of California, Berkeley, CA) and Tree view.

## Results

### Patients and tumors

The population studied was typical for bladder cancer. Most patients were male; the average age was 66 years (66 ± 11), and most had a history of cigarette smoking. Around 2/3 of tumors were NMI (Table 1) and most were of low or moderate grade. Following treatment, recurrence was observed in 24 patients (23%) and progression to invasion or metastases in 15 (14%).

### Genetic analysis of tumors

**Figure 2:**
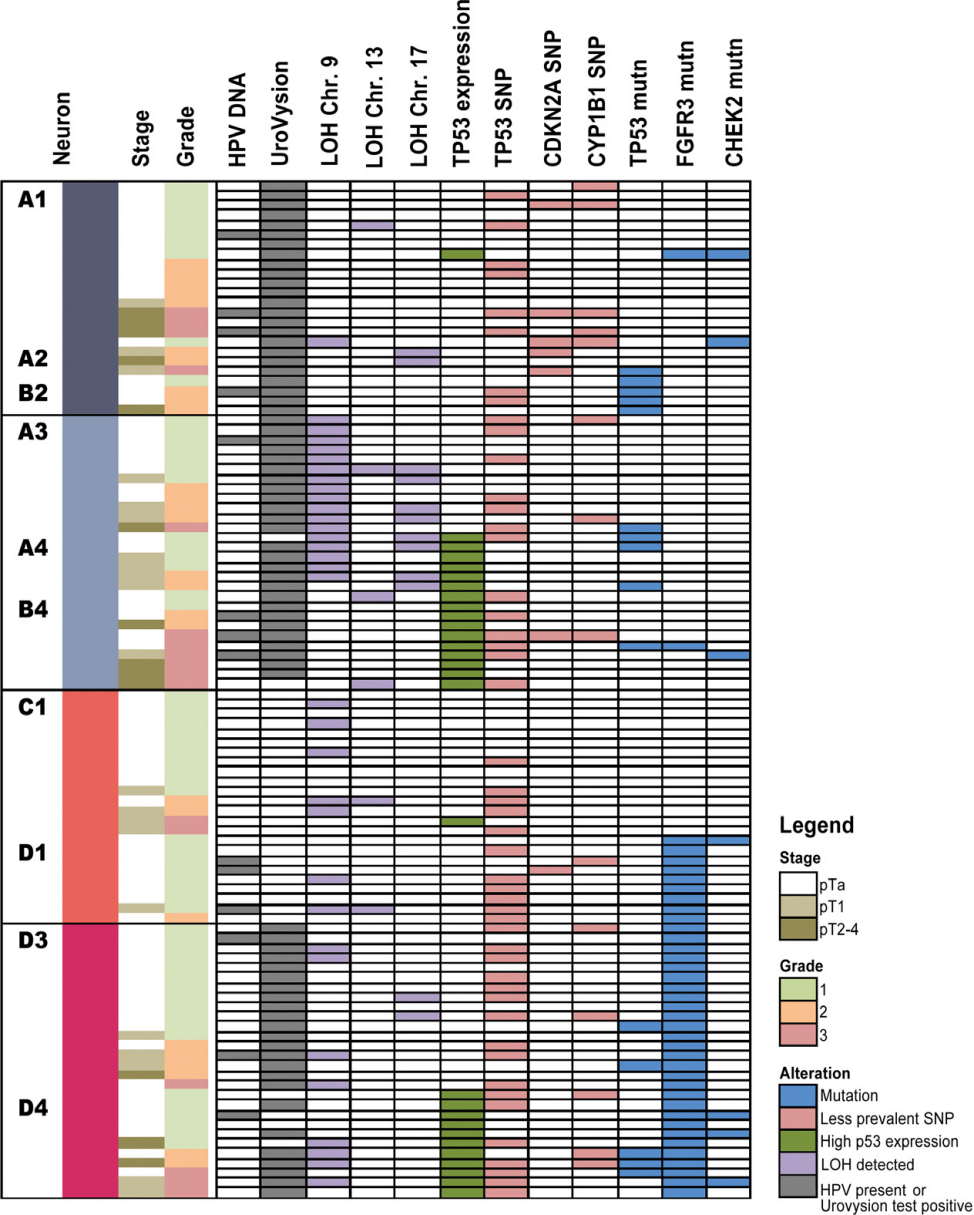
Pathological and molecular features of the individual tumors in this report. No patients were assigned to the nonlisted SOM neurons (B1, B3, C2-C4 and D2; see Fig. 4).

Genetic analysis revealed various molecular abnormalities (Table 1 and Fig.2). For example, mutations were found in *FGFR3* (*n* = 39, 38%), *TP53* (*n* = 15, 14%), and *CHEK2* (*n* = 7, 7%) tumors. Overall mutations were detected in 50 (48%) tumors, including 11 with more than one (six with *FGFR3* and *TP53*, and five with *FGFR3* and *CHEK2* mutations). Chromosomal loss was found in 41 (39%) tumors, including nine with more than one chromosome affected. While mutations of *CHEK2* (*n* = 7, 7%) and deletions of chromosome 13 (*n* = 6, 6%) were uncommon, the UroVysion test was positive in 74 (72%) samples.

### Progression from genetic markers

**Figure 3:**
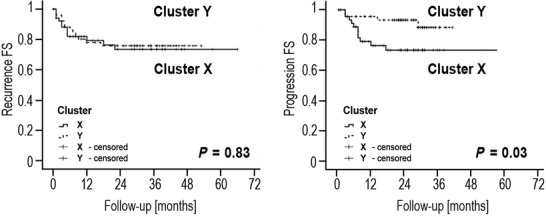
Recurrence and progression following treatment stratified using a self-organizing map.

The primary outcome for our study was disease progression to a more advanced stage. In univariable analysis, tumor stage (log rank *P* = 0.006) and grade (*P* < 0.001), HPV DNA (*P* < 0.004), Chromosome 9 (*P* = 0.04) and the A148T polymorphism (rs 3731249) in *CDKN2A* (*P* = 0.02) were associated with progression following treatment. Multivariable analysis of these parameters identified that tumor grade (Cox regression, *P* = 0.001, OR 2.9 (95% CI 1.6–5.2)) and the presence of HPV DNA (*P* = 0.017, OR 3.8 (95% CI 1.3–11.4) were the only independent predictors of progression. Unsupervised hierarchical clustering grouped the tumors into several branches (Figs.1 and 3). This approach did not significantly stratify progression free survival (log rank *P* = 0.39).

### Clusters

**Figure 4:**
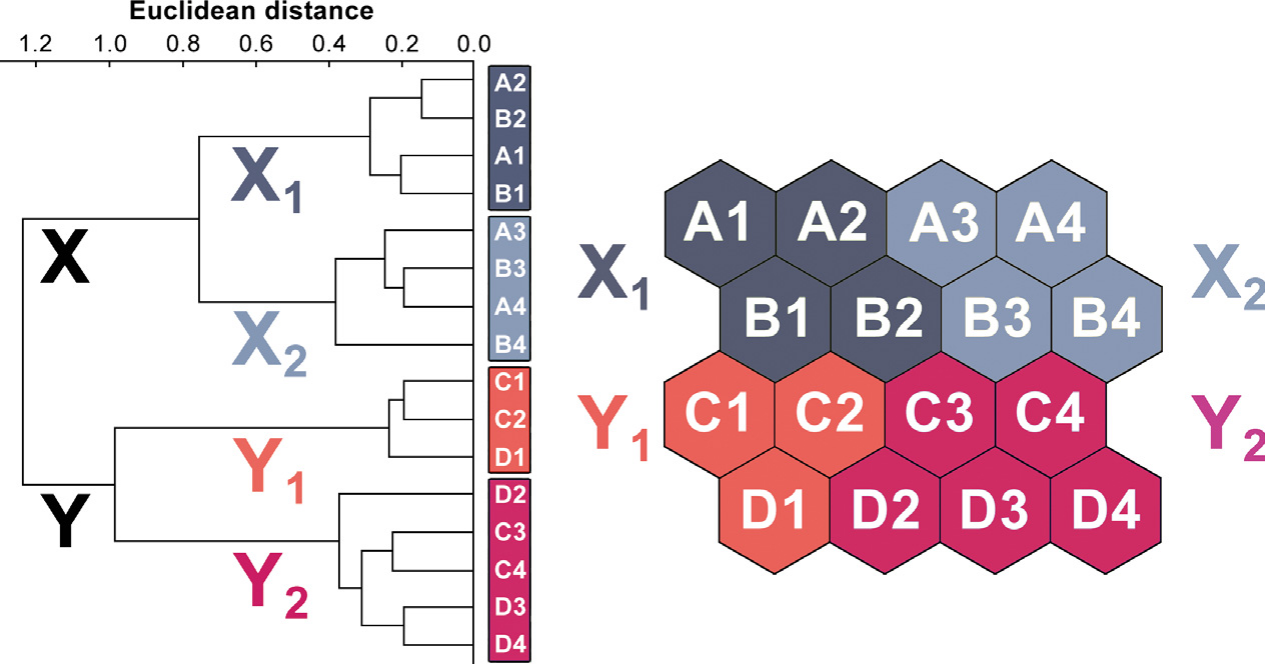
The output layer of the self organizing map applied. Clusters X, Y, and subclusters (X_1_, X_2_, Y_1,_ and Y_2_) of virtual patients and respective output neurons have been identified on the basis of the hierarchical cluster analysis.

**Figure 5:**
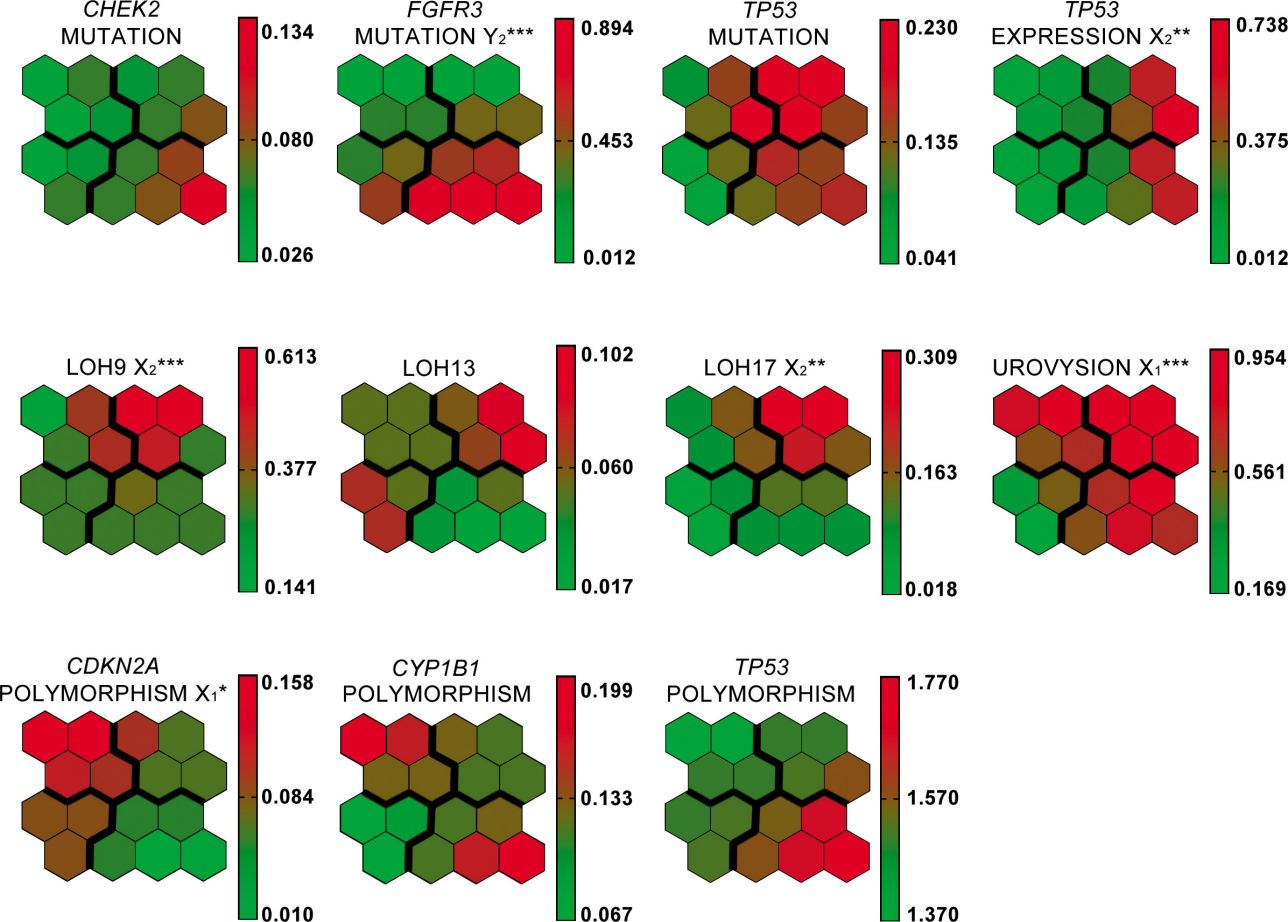
The associations (stronger if brighter red) of virtual patients' features with SOM regions. The intensity of colours is scaled independently for each variable. Variables with the same pattern over SOM are positively correlated. If the frequency of real patients with a given feature is significantly highest in any subcluster as compared to others, the symbol of the subcluster and the respective significance level (*P < 0.05; **P < 0.01; ***P < 0.001) are shown along with the variable name.

The two main clusters of SOM output neurons were distinguished: X and Y, each with a pair of sub-clusters: X_1_ and X_2_, and Y_1_ and Y_2_ (Fig.4). Patients with the worst prognosis were assigned to X_1_ and X_2_ (UroVysion test positive in 100% and 93%, respectively, and high frequency of *TP53* mutations, data in Table 2 and Fig. 2). The highest frequency of: (1) abnormal *TP53* expression (57%) and (2) heterozygocity loss for 9, 13 and 17 chromosome loci (71%) was recorded for patients in subcluster X_2_. In Y_1_ the UroVysion test was negative for all patients, and the *FGFR3* mutation ratio was quite high (38%). In Y_2_ the UroVysion test was positive in 86% patients and all of them had *FGFR3* gene mutation. These differences were also reflected in clinical variables (Table 2). Tumors with high grade and higher diameter were grouped mostly in subcluster X_1_ and X_2_. The highest ratio of recurrences (29%) was observed in subcluster Y_1_, where were only negative results of UroVysion test and none *TP53* mutations. Significant difference in frequency of patients with polymorphism A148T of *CDKN2A* gene, LOH frequency and *TP53* altered expression were observed between subclusters. Some other interesting observation can be noticed like negative correlation between *CDKN2A* and *TP53* gene polymorphism Figure5 is showing SOM component planes used to form the rules and their values with scale (down right-hand side of each histogram); components describe various characteristics of the subclusters.

**Table 2:** Results of the Kohonen SOM classifier (ns, not significant, *Kruskal–Wallis, **Tichý and Chytrý)

Variables	Subcluster	Subcluster	Subcluster	Subcluster	*P* value
	X_1_	X_2_	Y_1_	Y_2_	
	*n* (%)	*n* (%)	*n* (%)	*n* (%)	
Total No of patients	24 (23.0)	28 (27.0)	24 (23.0)	28 (27.0)	
Mean age	64	75.5	70.5	69	
No female	2	4	5	1	
Grade					
G1	10 (41.7)	13 (46.4)	19 (79.2)	18 (64.3)	<0.001*
G2-3	14 (58.3)	15 (53.6)	5 (12.5)	10 (21.4)	
Stage					
Ta	16 (66.7)	15 (53.6)	19 (79.2)	20 (71.4)	ns*
T1	3 (12.5)	8 (28.6)	5 (20.8)	5 (17.9)	
T2–T4	5 (20.8)	5 (17.8)	0	3 (10.7)	
Smoking history					
Current and ex	24 (100)	26 (92.9)	22 (91.7)	27 (96.4)	ns**
Never	0	2 (7.1)	2(8.3)	1 (3.6)	
Occupational exp.					
Yes	9 (37.5)	9 (32.1)	9 (37.5)	12 (42.9)	ns**
No	15 (62.5)	19 (67.9)	15 (62.5)	16 (57.1)	
No of tumors					
1	18 (75.0)	18 (64.3)	17 (63.0)	21 (75.0)	ns*
>1	6 (25.0)	10 (35.7)	7 (37.0)	7 (25.0)	
Tumor diameter					
2 cm	10 (41.7)	7 (25.0)	19 (79.2)	18 (64.3)	<0.001*
>2 cm	14 (58.3)	21 (75.0)	5 (20.8)	10 (35.7)	
Local recurrence					
Yes	5 (21.0)	7 (25.0)	7 (29.2)	5 (17.9)	ns**
No	19 (79.0)	21 (75.0)	17 (70.8)	23 (82.1)	
HPV infection					
Yes	4 (16.6)	4 (14.3)	3 (12.5)	3 (10.7)	ns**
No	20 (83.4)	24 (85.7)	21 (87.5)	25 (89.3)	
*CHEK2* mutation					
Yes	2 (8.3)	1 (3.6)	1 (4.2)	3 (10.7)	ns**
No	22 (91.7)	27 (96.4)	23 (95.8)	25 (89.3)	
*FGFR3* mutation					
Yes	1 (4.2)	1 (3.6)	9 (37.5)	28 (100)	<0.001**
No	23 (95.8)	2**7** (96.4)	15 (62.5)	0	
*TP53* mutation					
Yes	5 (20.8)	5 (17.9)	0	5 (17.9)	ns**
No	19 (79.2)	23 (82.1)	24 (100)	23 (82.10	
*TP53* expression					
Altered	1 (4.2)	16 (57.1)	1 (4.2)	11 (39.3)	<0.01**
Normal	23 (95.8)	12 (42.9)	23 (95.8)	17 (60.7)	
LOH chromosome 9					
Yes	1 (4.2)	17 (60.7)	7 (37.0)	8 (28.6)	<0.001**
No	23 (95.8)	11 (39.3)	17 (63.0)	20 (71.4)	
LOH chromosome 13					
Yes	1 (4.2)	3 (10.7)	2 (8.3)	0	ns**
No	23 (95.8)	25 (89.3)	22 (91.7)	28 (100)	
LOH chromosome 17					
Yes	2 (8.3)	8 (28.6)	0	2 (7.1)	<0.01**
No	22 (91.7)	20 (71.4)	24 (100)	26 (92.9)	
UroVysion test					
Positive	24 (100)	26 (92.9)	0	24 (85.7)	<0.001**
Negative	0	2 (7.1)	24 (100)	4 (14.3)	
*CDKN2A* polymorphism					
Ala/Thr	5 (20.8)	1 (3.6)	1 (4.2)	0	<0.05**
Ala/Ala	19 (79.2)	27 (96.4)	23 (95.8)	28 (100)	
*CYP1B1* polymorphism					
355T/T	5 (20.8)	3 (10.7)	1 (4.2)	5 (17.9)	ns**
355T/T and T/G	19 (79.2)	25 (89.3)	23 (95.8)	23 (82.1)	
*TP53* polymorphism					
Arg	16 (66.6)	15 (53.6)	13 (54.2)	11 (39.3)	ns**
Arg/Pro	7 (29.2)	12 (42.9)	9 (37.5)	13 (46.4)	
Pro	1 (4.2)	1 (3.5)	2 (8.3)	4 (14.3)	

## Discussion

Our knowledge of the molecular changes in BC has considerably grown over recent years 25. Currently, a number of conventional clinicopathological factors are useful in predicting survival of bladder cancer patients. These include tumor grade, stage, type, size, the presence of concomitant carcinoma in situ, patient age, tumor location, and presence of multiple tumors 26. As yet, there are no criteria that robustly predict the clinical outcome for individual patients with BC. Improvements in prediction may be made by the gain of information (e.g., through molecular biology) or by alternate methods of analysis. With this in mind, we have undertaken this study to evaluate the ability of SOM to integrate clinical–molecular information for stratifying outcomes in BC. Traditionally, statistical techniques such as Cox's proportional hazards and logistic regression are usually employed when analyzing prognostic information. Classic statistical modeling requires the explicit assumption of certain relationships within the data that are often unproven. ANNs offer a number of theoretical advantages, including ability to detect complex nonlinear relationships between variables, ability to detect all possible interactions between predictor variables, and the availability of multiple training algorithms 27. The ANN techniques depicted in the literature can be mainly categorized under two headings: supervised and unsupervised. Kohonen SOM consists in a feed forward neural network that uses an unsupervised training (partitional clustering). It means that, the data are directly divided into a set of clusters without any regard to the relationships between the clusters. These methods try to maximize some measure of similarity within the units (patients) of each cluster, while minimizing the similarity between clusters 28. SOM is combination of partitional clustering and projection methods. It can be used at the same time both to reduce the amount of data by clustering and to construct nonlinear projection of the data onto a low-dimensional display. In contrast to other clustering methods, the units in SOM become organized in such a way that nearby units on the gird are similar to another. The topology of the gird can be anything but in practice rectangular two-dimensional girds are preferred as they are easy to display 28,29.

In managing patients with BC, one of the principal problems for the clinician is prediction tumor recurrence and progression. It is likely that a combination of clinical, pathological, and molecular data are needed to optimize these outcome predictions. The future of molecular biomarkers in BC undoubtedly lies in of panels of markers that represent high- and low-grade disease. Examples of these include *FGFR3* and *TP53* mutations that are associated with a better or worse prognosis, respectively 30. Additional genetic changes that reflect underlying malignant traits, such as numerical chromosomal alternations from genetic instability, are useful as they identify global patterns within a disease rather than focusing upon specific events 31. In this work, we included many of these changes in an attempt to genotype tumors. In 2010, Catto et al. and Kim et al. identified six and eight progression-related genes in BC from microarray and either neurofuzzy modeling or hierarchical clustering, respectively 32,33. Of interest, the genes in these panels do not overlap, as found in other cancers 34,35. Here, we used SOM to explore a similar clinical scenario. We found that the SOM was easily understood by the clinician and could cluster tumors according to future clinical outcomes. SOMs appear to do this better than more traditional statistical analyses. In clinical care, this stratification could identify aggressive tumors needing early radical treatment and indolent ones suitable for less intense surveillance. The potential for SOMs is in real time help to guide patient choices. For example, in breast cancer detection, an unsupervised ANN model improved diagnosing performance when compared to classical feed-forward neural networks like multilayer perceptron (MLP), radial basis function (RBF), and probabilistic neural networks (PNN) 36,37. Thus, it is possible that SOMs could be integrated into patient pathways and used to guide their surveillance frequency or even treatment intent. There are a number of limitations to our work. For example, the analysis was based on a relatively low number of patients with a low event rate (number of cases with progression). However, we analysed a large number of genetic events that are known to characterize distinct BC molecular pathways, and as such, this work represents the first in BC to integrate clinical, molecular, and environmental prognostic biomarkers.

## Conclusions

We have shown that Kohonen SOM could cluster homogenous tumors according to genotype and that this stratified clinical outcomes when analyzed. SOMs are easy to understand and potentially outperform traditional statistical analyses. As such, their use needs more evaluation but they could potentially offer a real-time solution to integrating molecular data into patient pathways.

## References

[1] Thomas F, Rosario DJ, Rubin N, Goepel JR, Abbod MF, Catto JW (2012). The long-term outcome of treated high-risk nonmuscle-invasive bladder cancer: time to change treatment paradigm?. Cancer.

[2] Dudziec E, Goepel JR, Catto JWF (2011). Global epigenetic profiling in bladder cancer. Epigenomics.

[3] van Rhijn BW, van der Kwast TH, Vis AN, Kirkles WJ, Boeve ER, Jobsis AC (2004). FGFR3 and P53 characterize alternative genetic pathways in the pathogenesis of urothelial cell carcinoma. Cancer Res.

[4] Netto GJ (2012). Molecular biomarkers in urothelial carcinoma of the bladder: are we there yet?. Nat. Rev. Urol.

[5] Goebell PJ, Knowles MA (2010). Bladder cancer or bladder cancers? Genetically distinct malignant conditions of the urothelium. Urol. Oncol.

[6] Yates DR, Rehman I, Abbod MF, Meuth M, Cross SS, Linkens DA (2007). Promoter hypermethylation identifies progression risk in bladder cancer. Clin. Cancer Res.

[7] Catto JW, Miah S, Owen HC, Bryant H, Myers K, Dudziec E (2009). Distinct microRNA alterations characterize high- and low-grade bladder cancer. Cancer Res.

[8] Zieger K, Marcussen N, Borre M, Orntoft TF, Dyrskjot L (2009). Consistent genomic alterations in carcinoma in situ of the urinary bladder confirm the presence of two major pathways in bladder cancer development. Int. J. Cancer.

[9] Catto JWC, Abbod MF, Linkens D, Hamdy FC (2006). Neuro-fuzzy modeling: an accurate and interpretable method for predicting bladder cancer progression. J. Urol.

[10] Abbod MF, Catto JW, Linkens DA, Hamdy FC (2007). Application of artificial intelligence to the management of urological cancer. J. Urol.

[11] Kohonen T (1982). Self-organized formation of topologically correct feature maps. Biol. Cybern.

[12] Mostofi K, Sobin LH (1973). Histological typing of urinary bladder tumors. International histological classification of tumors.

[13] Chomczyński P, Sacchi N (1987). Single step method of RNA isolation by acid guanidinium thiocyanate-phenol-chloroform extraction. Anal. Biochem.

[14] Pisarek H, Stepień T, Kubiak R, Borkowska E, Pawlikowski M (2009). Expression of somatostatin receptor subtypes in human thyroid tumors: the immunohistochemical and molecular biology (RT-PCR) investigation. Thyroid Res.

[15] Borkowska E, Binka-Kowalska A, Constantinou M, Nawrocka A, Matych J, Kałużewski B (2007). P53 mutations in urinary bladder cancer patients from Central Poland. J. Appl. Genet.

[16] Borkowska E, Traczyk M, Pietrusiński M, Matych J, Kałużewski B (2010). The significance of *P53* codons 72 and 213 polymorphisms in urinary bladder cancer in Central Poland. Cent. European J. Urol.

[17] Borkowska E, Jędrzejczyk A, Kruk A, Pietrusiński M, Traczyk M, Rożniecki M (2011). Significance of *CDKN2A* gene A148T variant in patients with bladder cancer. Cent. European J. Urol.

[18] Cybulski C, Wokołorczyk D, Huzarski T, Byrski T, Gronwald J, Górski B (2007). A deletion in CHEK2 of 5,395 bp predisposes to breast cancer in Poland. Breast Cancer Res. Treat.

[19] Traczyk M, Borkowska E, Jędrzejczyk A, Pietrusiński M, Rożniecki M, Marks P (2011). Detection of loss of heterozygosity in patients with urinary bladder carcinoma: neoplastic tissue vs. urine sediment cells. Cent. European J. Urol.

[20] Bedoya D, Novotny V, Manolakos ES (2009). Instream and offstream environmental conditions and stream biotic integrity. Importance of scale and site similarities for learning and prediction. Ecol. Model.

[21] Ward JH (1963). Hierarchical grouping to optimize an objective function. J. Am. Stat. Assoc.

[22] Vesanto J, Himberg J, Alhoniemi E, Parhankangas J (2000). SOM Toolbox for Matlab 5.

[23] Vesanto J, Alhoniemi E (2000). Clustering of the Self-Organizing Map. IEEE Trans. Neural Netw.

[24] McCune B, Mefford MS (2011). PC-ORD Multivariate Analysis of Ecological Data, Version 6.06. MjM Software Design.

[25] van Rhijn B, Burger M, Lotan Y, Solsona E, Stief CG, Sylwester RJ (2009). Recurrence and progression of disease in non-muscle-invasive bladder cancer: from epidemiology to treatment strategy. Eur. Urol.

[26] van Rhijn BW, van Leenders GJ, Ooms BC, Kirkels WJ, Zlotta AR, Boeve ER (2010). The pathologist's mean grade is constant and individualizes the prognostic value of bladder cancer grading. Eur. Urol.

[27] Budayan C, Dikmen I, Birgonul MT (2009). Comparing the performance of traditional cluster analysis, self-organizing maps and fuzzy C-means method for strategic grouping. Expert Syst. Appl.

[28] Kohonen T (2013). Essentials of the self-organizing map. Neural Netw.

[29] Du KL (2010). Clustering: a neural network approach. Neural Netw.

[30] Cheng L, Zhang S, MacLennan GT, Williamson SR, Lopez-Beltran A, Montironi R (2011). Bladder cancer: translating molecular genetic insights into clinical practice. Hum. Pathol.

[31] Hernández S, López-Knowles E, Lloreta J, Kogevinas M, Jaramillo R, Amoros A (2005). FGFR3 and Tp53 mutations in T1G3 transitional bladder carcinomas: independent distribution and lack of association with prognosis. Clin. Cancer Res.

[32] Catto JWF, Abbod MF, Wild PJ, Linkens DA, Pilarsky C, Rehman I (2010). The application of artificial intelligence to microarray data: identification of a novel gene signature to identify bladder cancer progression. Eur. Urol.

[33] Kim WJ, Kim EJ, Kim SK, Kim YJ, Ha YS, Jeong PP (2010). Predictive value of progression-related gene classifier in primary non-muscle invasive bladder cancer. Mol. Cancer.

[34] Nobili S, Bruno L, Landini I, Napoli C, Bechi P, Tonelli F (2011). Genomic and genetic alterations influence the progression of gastric cancer. World J. Gastroenterol.

[35] Cancer Genome Atlas Network (2012). Comprehensive molecular portraits of breast tumours. Nature.

[36] Belciug S, Gorunescu F, Gorunescu M, Salem ABM (2007).

[37] Markey MK, Lo JY, Tourassi GD, Floyd CE (2003). Self-organizing map for cluster analysis of a breast cancer database. Artif. Intell. Med.

